# Three-Dimensional Printing of Personalized Carbamazepine Tablets Using Hydrophilic Polymers: An Investigation of Correlation Between Dissolution Kinetics and Printing Parameters

**DOI:** 10.3390/polym17152126

**Published:** 2025-08-01

**Authors:** Lianghao Huang, Xingyue Zhang, Qichen Huang, Minqing Zhu, Tiantian Yang, Jiaxiang Zhang

**Affiliations:** 1 Key Laboratory of Marine Drugs, Ministry of Education, School of Medicine and Pharmacy, Ocean University of China, Qingdao 266003, China; 2 Pharmaceutical Products Research and Development Center, Marine Biomedical Research Institute of Qingdao, Qingdao 266137, China; 3 Material Characterization, Thermo Fisher Scientific, Shanghai 201203, China

**Keywords:** hot-melt extrusion, FDM-3D printing parameter, Box–Behnken design, carbamazepine, amorphous solid dispersion

## Abstract

Background: Precision medicine refers to the formulation of personalized drug regimens according to the individual characteristics of patients to achieve optimal efficacy and minimize adverse reactions. Additive manufacturing (AM), also known as three-dimensional (3D) printing, has emerged as an optimal solution for precision drug delivery, enabling customizable and the fabrication of multifunctional structures with precise control over morphology and release behavior in pharmaceutics. However, the influence of 3D printing parameters on the printed tablets, especially regarding in vitro and in vivo performance, remains poorly understood, limiting the optimization of manufacturing processes for controlled-release profiles. Objective: To establish the fabrication process of 3D-printed controlled-release tablets via comprehensively understanding the printing parameters using fused deposition modeling (FDM) combined with hot-melt extrusion (HME) technologies. HPMC-AS/HPC-EF was used as the drug delivery matrix and carbamazepine (CBZ) was used as a model drug to investigate the in vitro drug delivery performance of the printed tablets. Methodology: Thermogravimetric analysis (TGA) was employed to assess the thermal compatibility of CBZ with HPMC-AS/HPC-EF excipients up to 230 °C, surpassing typical processing temperatures (160–200 °C). The formation of stable amorphous solid dispersions (ASDs) was validated using differential scanning calorimetry (DSC), hot-stage polarized light microscopy (PLM), and powder X-ray diffraction (PXRD). A 15-group full factorial design was then used to evaluate the effects of the fan speed (20–100%), platform temperature (40–80 °C), and printing speed (20–100 mm/s) on the tablet properties. Response surface modeling (RSM) with inverse square-root transformation was applied to analyze the dissolution kinetics, specifically t_50%_ (time for 50% drug release) and Q_4h_ (drug released at 4 h). Results: TGA confirmed the thermal compatibility of CBZ with HPMC-AS/HPC-EF, enabling stable ASD formation validated by DSC, PLM, and PXRD. The full factorial design revealed that printing speed was the dominant parameter governing dissolution behavior, with high speeds accelerating release and low speeds prolonging release through porosity-modulated diffusion control. RSM quadratic models showed optimal fits for t_50%_ (R^2^ = 0.9936) and Q_4h_ (R^2^ = 0.9019), highlighting the predictability of release kinetics via process parameter tuning. This work demonstrates the adaptability of polymer composite AM for tailoring drug release profiles, balancing mechanical integrity, release kinetics, and manufacturing scalability to advance multifunctional 3D-printed drug delivery devices in pharmaceutics.

## 1. Introduction

Precision medicine, in which medication or health care is offered individually according to a patient’s genetic characteristics, living environment, and daily lifestyle, has gained significant traction across the clinical and research domains [1]. Precision drug delivery by means of polymer-based controlled drug delivery, individualized formulation design, and additive manufacturing technologies is necessary to achieve precision medicine. Polymeric-based controlled release not only enhances drug efficacy and safety but also enables the selective delivery of drugs to specific organs, tissues, or cells at a predictable rate and through a well-understood mechanism [2]. The rapid evolution of pharmaceutical manufacturing technologies has significantly enhanced pharmaceutical research capabilities, enabling the creation of next-generation therapeutic products with superior efficacy and functionality compared with traditional formulations [3].

Recent breakthroughs in multi-omics analytics, AI-driven molecular modeling, and nanotechnology have collectively deepened our mechanistic understanding of disease pathogenesis and pharmacological responses at the molecular level [4,5,6,7]. This interdisciplinary progress has spurred the development of precision medicine strategies tailored to individual patient characteristics, including body mass index, chronological age, biological sex, and other physiological parameters [8]. Such personalized approaches are increasingly gaining traction across both academic research and industrial R&D sectors. This technological momentum has been particularly impactful in advancing precision medicine, in which the development of customizable drug delivery systems (DDSs) plays a central role. Currently, numerous technologies are available for achieving precise or personalized oral DDSs, including nanotechnology-based approaches, microfabrication techniques (microelectromechanical systems and microfluidic chips), and three-dimensional (3D) printing technology [9,10,11,12,13,14]. Among these technologies, 3D printing enables flexible drug release control by adjusting 3D structures, facilitating individualized and precise oral drug delivery [15,16].

Three-dimensional printing is a procedure that constructs objects layer-by-layer based on computer-aided designs, which allows for precise, patient-specific dose dispensing instead of fixed-dose formulations [17,18,19,20,21]. Printed tablets integrate multiple active substances and exhibit diverse forms and dimensions. Therefore, their release modalities can be adjusted to align with patient preferences by modifying the tablet shape and composition [22,23]. Doctors or caregivers can update the necessary dose instantly by observing the patients’ test outcomes [20]. This real-time dose adjustment is crucial for delivering personalized treatment customized to the patient’s current condition. The flexibility of printed tablets in terms of composition and form provides an additional level of customization, complementing dose adjustment by physicians and enhancing the overall precision and personalization of the treatment. The level of personalization is further supported by advancements in 3D printing technologies, which enable precise fabrication of pharmaceutical products. A variety of 3D printing techniques, such as stereolithography, selective laser sintering, selective laser melting, material extrusion, material jetting, inkjet-based methods, fused deposition modeling, binder deposition, and bioprinting, serve to accelerate the printing process [24,25,26,27,28,29,30,31]. Droplet deposition modeling is an emerging technology with significant promise for personalized pharmaceutical manufacturing [32]. Droplet deposition modeling (DDM) offers significant advantages for manufacturing pharmaceutical formulations containing thermally labile drug molecules because it eliminates the need for dual thermal cycles in upstream processing [33,34]. Among the seven distinct AM categories defined by the American Society for Testing and Materials (ATSM), fused deposition modeling (FDM) 3D printing has emerged as the most prevalent pharmaceutical AM technology [34,35]. As a widely used 3D printing method in pharmaceutical manufacturing, FDM-3D printing is popular because it is easy to use, affordable, precise, and can produce many items quickly [36].

Three-dimensional printing technology offers great flexibility in fabricating complex geometries with precise control over structural and functional properties, aligning seamlessly with the rising clinical need for personalized pharmaceuticals. The FDM-3D printing process usually involves three key stages: (1) digital model design using CAD or medical imaging to create a 3D geometry for the dosage form; (2) slicing, where the model is algorithmically divided into 2D layers to generate machine-readable G-code with layer parameters; and (3) additive manufacturing, during which a heated extruder melts drug-loaded filaments and deposits them layer-by-layer onto a platform to fabricate the designed structure. A number of key parameters of tablets’ structure have been studied extensively by Kocabas et al., Fanous et al., Chen et al., and Obeid et al. in the slicing process, including the infill density, wall line, and thickness [37,38,39,40]. By adjusting the infill density, printing patterns, and wall thickness, various functional tablets have been fabricated to meet diverse needs. However, the abovementioned investigations have focused solely on the impact of structural parameters during the slicing process on tablet performance, while neglecting systematic research on printing parameters, including the platform temperature, cooling rate, and printing speed. This may lead to poor understanding of their interactive effects on tablet quality. The platform temperature can influence layer-to-layer bonding and filament thermal stability during deposition, whereas the cooling rate affects the crystallinity of polymers and the mechanical properties of the final product [41,42]. The printing speed affects the uniformity of material deposition and the dimensions of the tablets. An incomplete understanding of how these parameters interact with each other directly leads to suboptimal precision and inefficiency in the manufacturing process [43]. Therefore, a systematic study of how these parameters interact and affect tablet quality is required to improve the FDM-3D printing process, making it more precise, efficient, and better for producing personalized drugs.

Response surface methodology (RSM) is one of the most common approaches for designing experiments and optimizing different environmental processes based on various statistical and mathematical techniques [44]. Box–Behnken design (BBD), an RSM design, can efficiently determine the nature of the response surface in an experimental region [44]. It requires fewer runs in a three-factor experimental design than other RSM designs, and avoids extreme treatment combinations. BBD can achieve a better process and product understanding by revealing the relationship between the input factors and response parameters. Many reports have demonstrated the successful application of optimized HME conditions in pharmaceutical research [45,46,47]. So, as a systematic experimental design methodology, RSM can offer an effective approach to address the challenges in FDM-3D printing parameter optimization. It enables systematic evaluation of how critical parameters like the platform temperature, printing speed, and fan speed interact to influence the tablet quality attributes such as the dimensional accuracy and mechanical strength. Based on the results derived from RSM, the optimal process conditions can be identified to enhance manufacturing precision, boost production efficiency, and develop personalized pharmaceutical products.

In this study, ASD filaments were prepared using HME technologies. The PLM, DSC, and PXRD results confirmed that the extrusion transformed into ASD after the extrusion process. According to the Box–Behnken design, fifteen CBZ tablets with different parameters were successfully prepared by 3D printing. Direct compressed tablets of physical mixtures (PMs) and extrudates (EXT) were prepared for comparison. In vitro studies showed that the dissolution rate and degree of extrudates were lower than those in the PM group. The dissolution profiles of 3D-printed CBZ tablets displayed variations owing to their different printing setups. This research sheds new light on the hitherto uninvestigated parameters within 3D printing and offers profound insights into precise drug delivery enabled by programmable release, potentially heralding a paradigm shift in both fields.

## 2. Materials and Methods

### 2.1. Materials

Carbamazepine (CBZ) was purchased from Macklin Biochemical Co., Ltd. (Shanghai, China) and exhibits a type III crystalline form with the highest stability at ambient temperature [48,49]. Hydroxypropyl methylcellulose acetate succinate (HPMC-AS) was purchased from Taian Ruitai cellulose Co., Ltd. (Taian, China), and HPC-EF was donated by Asland (Shanghai, China). All the remaining chemicals, solvents, and reagents employed in this study were of analytical or HPLC grade.

### 2.2. Methods

#### 2.2.1. Process Development of 3D-Printed Tablets

##### HME

**Figure 1:**
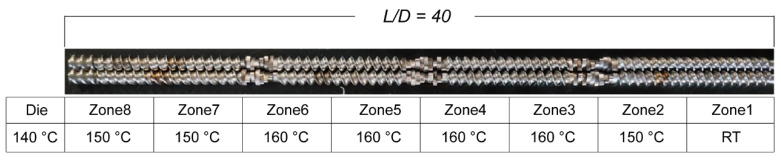
The demonstration of screw design and temperature setups.

CBZ, HPMC-AS, and HPC-EF were placed in a vacuum oven at 50 °C overnight to remove moisture prior to formulation. CBZ (30% *w*/*w*) was blended with HPMC-AS (60% *w*/*w*) and HPC-EF polymers (10% *w*/*w*) and then marked as a physical mixture (PM). A Thermal Fisher Scientific 11 mm corotating twin-screw extruder (Process 11 HYG) (Thermal Fisher Scientific, Shanghai, China) was employed to prepare the CBZ-loaded filaments. The eight zones in the barrel section of the extruder can be heated or cooled individually, whereas only a separated die section can be heated. A 2 mm pole insert was used to re-shape the extruded filaments with a diameter around 1.75 mm. An automated feeder (Thermo Fisher Scientific, Shanghai, China) was used to deliver the PM to the feeding zone (zone 1), with the feeding rate adjusted to approximately 3 g/min. The screw configuration and extrusion temperature setup are shown in Figure 1. During the extrusion process, the melting temperature, torque, and die pressure were monitored to ensure a steady process. For the purpose of attaining filaments possessing a consistent diameter of 1.70 ± 0.05 mm, a conveyor belt (Thermo Fisher Scientific, Shanghai, China) was utilized to rectify and collect the extruded filaments. Only after the torque and die pressure reached a stable condition (fluctuation of less than 5%) were the filaments collected and stored in a validated desiccator for future evaluation and FDM-3D printing. The extruded filaments were powdered using a granulator and then sieved through an ASTM 40-mesh sieve. The sieved particles were marketed as EXT for subsequent characterization studies.

##### Three-Dimensional Printing

**Figure 2:**
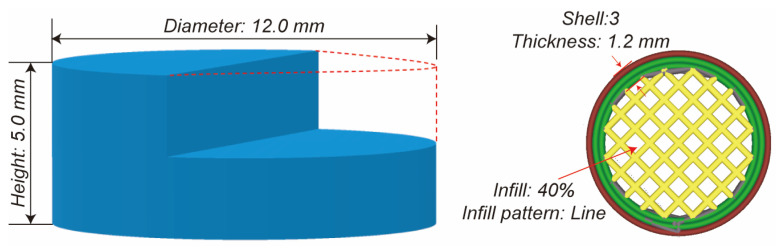
The demonstration of the 3D design and the design parameters of the tablets.

Digital tablet prototypes were computationally modeled using 3D construction software (3D Builder, Version 18.0.1931.0, Microsoft Corporation, Redmond, Washington, USA) with cylindrical geometric specifications. The tablet was designed as a cylinder with a diameter of 12.0 mm and height of 5.0 mm. As illustrated in Figure 2, these virtual models were sliced using Cura software (Version 5.2.1, Ultimaker, Utrecht, The Netherlands) with parameter customization, including shell dimensions, vertical resolution, and internal matrix density. Critical manufacturing parameters included the elimination of surface layers during slicing protocols. The designed tablets were printed using a fused deposition modeling 3D printer (Ender-3 S1 Pro, Shenzhen Creality 3d Technology Co., Ltd., Shenzhen, China) with a 0.4 mm deposition nozzle, while other printing parameters including the extrusion temperature, platform heating, and deposition velocity are shown in Table 1. The 3D-printed tablet was fabricated with a three-layer shell structure achieving a total thickness of 1.2 mm (0.4 mm per layer), utilizing a rectilinear infill pattern at 40% volumetric density to balance mechanical stability and controlled porosity (Figure 2). The extrusion nozzle temperature was precisely maintained at 200 °C (±1 °C calibration accuracy) throughout the printing process to ensure optimal polymer flow and structural integrity. Comparative conventional tablets (PM/EXT) were prepared through a single-punch hydraulic tablet press (Nuzhen Technology Co., Ltd., Shanghai, China) with standardized mass (500 mg) and compression force (100 bar).

##### Process Development Using Design of Experiments (DoE)

Box–Behnken design response surface methodology was employed to investigate the effects of the printing speed, platform temperature, and fan speed on the dimensional parameters (diameter, height, mass) and dissolution profiles of CBZ 3D-printed tablets. Three independent variables—fan speed (F, %), platform temperature (T, °C), and printing speed (S, mm/s)—were investigated, with five dependent variables including the tablet diameter (D), height (H), mass (M), accumulated CBZ released at 4 h dissolution (Q_4h_), and time to 50% w/w drug released (t_50%_). The reproducibility was also investigated by three replicates conducted at the central point (R2, R3, R5 in Table 1).

#### 2.2.2. Characterization of Raw Materials, Intermediate Extrudates, and Printed Tablets

##### Thermalgravimetric Analysis (TGA)

The thermal degradation behavior of CBZ, HPMC-AS, and PM was investigated using TGA (Netzsch TG209F3, NETZSCHGeratebau GmbH, Selb, Bavaria, Germany). Amounts of 5–10 mg of the samples were precisely weighted and then loaded into aluminum crucibles and subjected to controlled heating from 35 °C to 400 °C under a continuous high-purity nitrogen atmosphere (flow rate: 60 mL/min). Continuous mass loss measurements were recorded at a heating rate of 10 °C/min to monitor the thermal decomposition profiles. All experimental data were acquired through the Proteus^®^ Analysis software suite (Version 8.17) and subsequently processed using Microsoft Excel (Version 2310, Build 16.0.16924.20054) for comprehensive thermal stability evaluation.

##### Differential Scanning Calorimetry (DSC)

The thermal behavior of CBZ, HPMC-AS, PM, and EXT was characterized using differential scanning calorimetry (DSC 200 F3, NETZSCH Gerätebau GmbH, Bavaria, Germany). Precisely weighed samples (5–15 mg) were encapsulated in hermetically sealed aluminum crucibles (DSC Consumables Inc., Austin, MN, USA) using standardized crimping protocols. All samples underwent linear heating from 30 °C to 220 °C at 20 °C/min under nitrogen purge (50 mL/min). Thermograms were acquired through Proteus^®^ Thermal Analysis software with subsequent data processing performed in Microsoft Excel (v2310 Build 16.0.16924.20054) for thermodynamic parameter calculation and graphical representation.

##### Powder X-Ray Diffraction (PXRD)

The crystallographic characteristics of CBZ, HPMC-AS, PM, and EXT were systematically analyzed using a high-resolution powder X-ray diffractometer (PXRD, D/max-2200PC, Rigaku Corp., Tokyo, Japan). Samples were precisely mounted in low-background silicon holders and subjected to Bragg–Brentano geometry scanning with Cu Kα radiation (λ = 1.5406 Å). The diffraction patterns were acquired under optimized operational parameters (45 kV tube voltage, 15 mA filament current) across an angular range of 5–50° 2θ with controlled scanning conditions: 2°/min scan rate, 0.02° step size, and 0.0025° angular resolution. For comparative crystallinity assessment, raw diffraction data were processed through Jade 6.0 software (Materials Data Inc., Livermore, CA, USA) with subsequent background subtraction and Kα2 stripping. Final comparative diffractogram overlays (intensity vs. 2θ) were generated for quantitative phase analysis and polymorphic form identification.

##### Hot-Stage Polarized Light Microscopy (PLM)

A polarizing photomicroscope system (CX40P, Ningbo ShunYu Analytical Instrument Co., Ltd., Yuyao, China) coupled with a thermal analysis stage was employed to examine the thermal transition characteristics and residual crystalline CBZ in processed formulations. The initial heating temperature was set at 35 °C, and the samples were heated at a rate of 20 °C/min until the melting point of CBZ was reached. Then, the samples were cooled down at room temperature. Samples were analyzed at 10× optical magnification under cross-polarized illumination conditions. Crystalline components in all tested formulations were confirmed through the detection of characteristic birefringence patterns. Digital image acquisition was performed using a scientific-grade CCD camera (EP-SUF880, Quest Scientific Instruments Inc., North Vancouver, BC, Canada) equipped with specialized optical filters, including a 530 nm retardation compensator (U-TP530, Olympus Optical) for enhanced contrast visualization under differential interference contrast conditions.

##### Qualification and Quantification of CBZ Using HPLC

CBZ calibration standards (0.025–0.4 mg/mL) were prepared via sonication of 40 mg CBZ in methanol followed by stepwise 1:2 dilutions. HPLC analysis was performed using an Agilent 1260 Infinity II system with a Diamonsil^®^ Plus C18 column (250 × 4.6 mm, 5 µm) and a mobile phase of water–acetonitrile–methanol (30:10:60 *v*/*v*/*v*) at 1.0 mL/min. Detection at 286 nm was achieved using a DAD detector, with data processed by Agilent software (Version C.01.07 SR2 [255], Agilent Technologies, Inc., Santa Clara, CA, USA).

#### 2.2.3. In Vitro Drug Release Study

Drug release profiles of the tablets were evaluated using a U.S. Pharmacopeia (USP apparatus II)-compliant dissolution apparatus (RC8MD, TIANDA TIANFA, Tianjin, China). Dissolution testing was performed in triplicate following USP standards using Simulated Intestinal Fluid (USP SIF, pH 6.8) prepared from 0.025 M KH_2_PO_4_ and Na_2_HPO_4_ buffer without pancreatin. The 900 mL dissolution medium was maintained at 37 ± 0.5 °C under paddle agitation at 100 rpm for 12 h. Samples were collected at 5, 15, 30, 60, 120, 240, 360, 480, 600, and 720 min post-immersion. Quantification of released CBZ was conducted via HPLC analysis; detailed experimental methods have been mentioned in Section 2.2.2.

## 3. Results and Discussion

### 3.1. Thermal Stability

**Figure 3:**
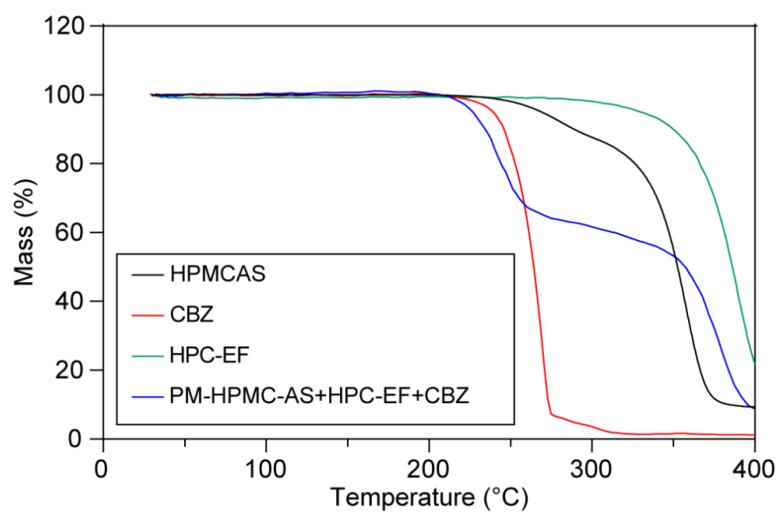
The thermogravimetric curves of raw CBZ, HPMC-AS, HPC-EF, and PM.

The thermal stability of materials is critical during thermal processes like HME or FDM-3D printing, so thermalgravimetric studies were conducted to measure the weight loss with the ramp. As shown in Figure 3, TGA curves revealed single-stage degradation for CBZ, HPMC-AS, and HPC-EF, with decomposition initiation temperatures exceeding 230 °C. It is interesting that the PM showed two-staged thermal degradation with about 40% weight loss from 210 to 230 °C and another 50% weight loss from 340 to 400 °C. The first stage of degradation might be because of the degradation of CBZ itself as well as the interaction between CBZ and HPMCAS or HPC, because such weight loss (around 40%) occurs very close to the degradation temperature of CBZ itself and also exceeds the drug loading of CBZ (30%) [50]. The second-stage degradation might be mainly attributed to the degradation of the remaining HPC and HPMCAS [51]. Such thermal degradation behavior offered significant information that both thermal process temperatures should not exceed 210 °C. So, the HME was conducted at 160 °C and 3D printing was conducted at 200 °C to avoid potential thermal degradation during manufacturing.

### 3.2. Preparation of 3D Printable Filaments

Initially, the formulation was set as CBZ: HPMCAS = 3:7 (*w*/*w*). However, the filaments prepared with this formulation showed poor performance, specifically frequent fracture during the 3D printing process. Based on our previous research, it was found that the addition of HPC-EF could effectively improve the mechanical properties of the filaments [18]. Therefore, after systematic optimization, the final formulation was determined to be a ternary blend of CBZ, HPMC-AS, and HPC-EF at a weight ratio of 3:6:1 (*w*/*w*/*w*).

A ternary blend of CBZ, HPMC-AS, and HPC-EF (3:6:1 *w*/*w*/*w*) was mixed thoroughly using a mortar and pestle before extrusion. Process parameters including torque and die pressure were monitored during operation. Filament collection began after achieving steady-state conditions (torque/pressure fluctuations ≤ 5%). Thermal expansion was observed as extrudates exited the die. A conveyor system with adjusted speed was utilized to maintain filament straightness and diameter uniformity. Final filament diameter was controlled to 1.70 ± 0.05 mm for compatibility with 3D printer feed mechanisms. Notable physical changes were observed during the extrusion process where the extrudates developed yellow color alongside a transition from opaque to transparent compared to the PM. The yellow coloration of EXT could be attributed to the formation of extended π-conjugated structures, as reported by the authors of [52,53]. Furthermore, the transparency indicates that crystalline CBZ was amorphized and dispersed into the HPMC-AS and HPC-EF polymer matrix that formed ASD [54].

### 3.3. Solid-State Analysis

#### 3.3.1. Characterization of Thermal Behavior of Raw Materials and Filaments

**Figure 4:**
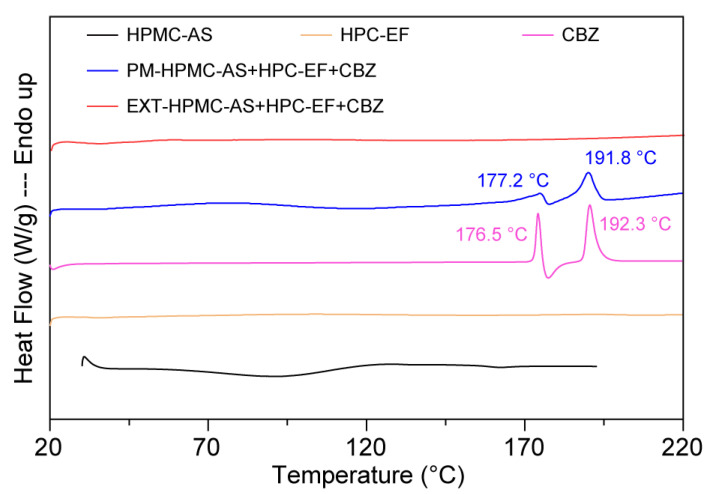
The DSC curves of raw materials, physical mixtures, and HME extrudates.

DSC was employed to characterize the crystalline states of pure CBZ, HPMC-AS, HPC-EF, PM, and EXT. CBZ possessed five polymorphic forms (I–V) and Form III has been proved thermodynamically stable at ambient temperature [48,49]. As shown in Figure 4, pure CBZ displayed two distinct endothermic events at 192.3 °C, corresponding to the polymorphic transition from Form III to Form I (around 170 °C) followed by melting of Form I at around 193 °C [55]. This thermal behavior is characteristic of CBZ Form III and can be used to identify the crystalline CBZ in PM and EXT. The tested HPMC-AS exhibited a glass transition temperature (Tg) of ~110 °C, consistent with the literature reports of Tg ranging from 90 to 122 °C depending on vendor/grade [52].

The PM showed attenuated melting peaks at 177.2 °C and 191.8 °C. The decrease in the fusion enthalpy of CBZ in the PM group compared to pure CBZ (~177 °C:48.4 → 39.3 J/g; ~193 °C:104.6 → 64.7 J/g) may be related to potential interactions between CBZ’s amide groups and the hydroxyls of HPMC-AS/HPC-EF, though insufficient to fully disrupt crystalline domains [56]. Notably, the missing detectable endothermic peaks in the EXT samples suggested potential amorphization of CBZ during processing. However, the absence of thermal transitions must be interpreted cautiously: (1) DSC detection limits (~1–2% crystallinity) may fail to detect minor ordered regions; (2) high polymer content could mask drug-specific thermal events; and (3) rapid cooling during extrusion may generate metastable amorphous domains prone to recrystallization during storage. Subsequent PXRD and PLM analyses (discussed in the following sections) will provide complementary insights into crystallinity changes in extruded formulations.

#### 3.3.2. Hot-Staged PLM of Raw Materials and Filaments

**Figure 5:**
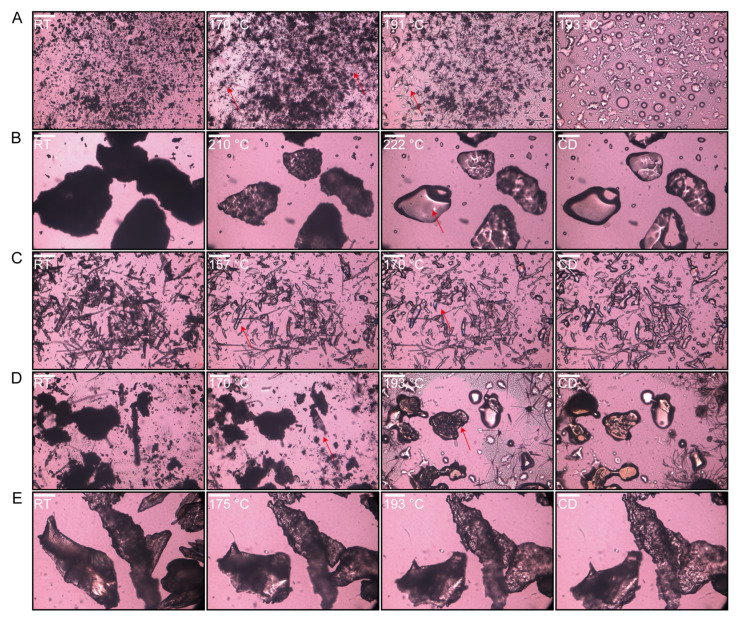
Hot-stage PLM pictures of each heat formulation until melted and cooled down (CD): (**A**) CBZ; (**B**) HPMC-AS; (**C**) HPC-EF; (**D**) PM; (**E**) EXT. Scale bar: 50 µm.

The crystalline state of CBZ within extruded filaments was investigated using PLM. At room temperature, CBZ existed as a stable crystalline solid, predominantly in Form III [57]. When heated to 170 °C, CBZ started a crystal form transformation, yielding needle-like crystals (Figure 5A). Then, CBZ initiated melting at 192 °C. According to the previously published literature, there is a polymorphic transformation of Form III CBZ at around 170 °C to Form I and then melting at around 190 °C, which matched with the DSC curves [57]. This thermal behavior aligns with the compound’s known melting characteristics, confirming its crystalline integrity prior to extrusion [58]. HPMC-AS and HPC-EF displayed homogeneous morphology under PLM, consistent with their amorphous and isotropic nature. During the heating process of the polymers, a texture change (gradual transition from opaque to transparent) was observed, which may relate to the glass transition identified by DSC. Additionally, the HPMCAS and HPC-EF showed complete softening and flow transitions at approximately 222 °C and 170 °C, respectively (Figure 5B,C).

When the PM was heated, a crystal transformation phenomenon occurred at around 170 °C, while the melting of HPC-EF could not be observed at 170 °C because of less content (Figure 5D). Meanwhile, HPMC-AS remained intact throughout. Upon reaching 193 °C, as CBZ completely melted, the three components started to form an ASD. Subsequent to cooling, they attained a state closely similar to that of the EXT. The EXT exhibited a complete absence of birefringence under cross-polarized light (Figure 5E), indicating the absence of crystalline CBZ domains larger than 50 μm. This is consistent with ASD formation, where HME-induced molecular dispersion of CBZ within HPMC-AS/HPC-EF eliminates micron-scale crystallinity. Although DSC and PLM collectively support ASD formation, definitive proof of crystalline-to-amorphous transformation requires PXRD analysis to detect residual crystallinity at the atomic level.

#### 3.3.3. Characterization of Crystalline States of Raw Materials and Filaments

**Figure 6:**
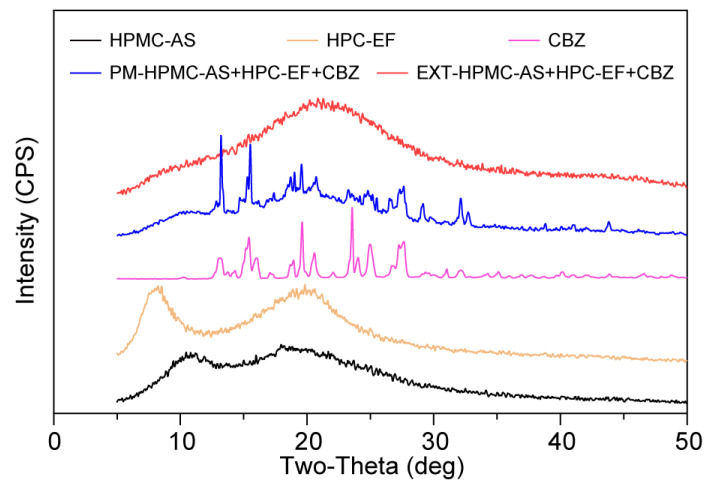
PXRD curves of HPMC-AS, HPC-EF, CBZ, PM, and EXT.

The solid-state characteristics of the formulation components were analyzed using PXRD, a technique providing atomic-level insights into crystallinity [59]. Crystalline CBZ exhibited characteristic diffraction peaks at 2θ of 13.3°, 15.4°, 19.7°, and 25°, consistent with the literature reports (Figure 6) [57]. The above characteristic peaks cannot be observed in EXT, indicating molecular-level dispersion of CBZ within the HPMC-AS and HPC matrix to form ASD by HME. The absence of crystalline reflections in neat HPMC-AS and HPC-EF profiles further supported their amorphous nature. These PXRD results support the DSC and PLM findings, confirming successful CBZ amorphization during HME. The integrated use of solid-state analysis highlights their complementary roles in characterizing solid-state transformations, with PXRD providing definitive crystallinity assessment critical for validating ASD formation.

### 3.4. DoE Studies

#### 3.4.1. Physical Properties of 3D-Printed Tablets

A Box–Benken experimental design of fifteen groups (R1–R15) was conducted to investigate the correlation between the printing parameters and quality attributes of the printed tablets (Table 1). Printing parameters including the fan speed (F: 20–100%), platform temperature (T: 40–80 °C), and printing speed (S: 20–100 mm/s) were studied, and quality attributes including the tablets’ appearance, dimensions, weight variation, and dissolution performance were evaluated.

##### Morphology Study

**Figure 7:**
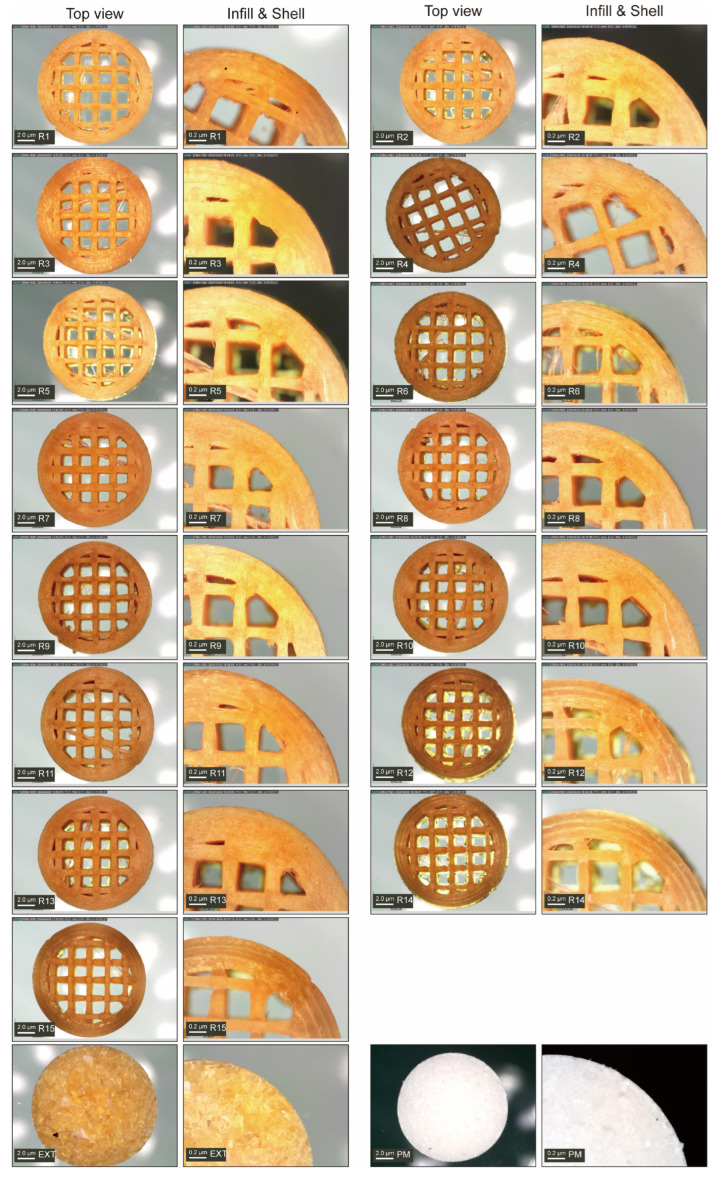
Structure diagram of CBZ tablets.

The morphology study was conducted by capturing the microscopic images of all fifteen groups of tablets, which showed a smooth surface and homogeneous components across all samples (Figure 7). Unlike the printed tablets, the microscopic images showed that the directly compressed PM and EXT tablets were composed of numerous small particles compressed together. Such a difference between printed and compressed tablets may result in differences in drug release performance. Interestingly, when the 3D printing speed was 20 mm/s, the shell layers of the tablets (R7–9 and 13) appeared less distinct under microscopic observation, whereas at a printing speed of 100 mm/s, clear layer structures were visible in the tablets (R6, 12, 14, and 15). This suggests that lower printing speeds may promote stronger interlayer fusion or excessive material accumulation at the layer interface, reducing the boundary between successive printing layers. Notably, increasing the fan speed failed to improve the quality of printing tablets at lower printing speeds. Slower nozzle movement might allow more time for material dispersion and partial melting at the shell, making layer boundaries less clear. In contrast, higher printing speeds could maintain sharper layer boundaries by reducing the interlayer interaction time, preserving the structural hierarchy of the printed tablets. These morphological differences induced by the printing speed are able to influence the mechanical properties and drug release kinetics, which will be further explored in the subsequent analysis.

##### Height and Diameter

As shown in Table 1, the designed diameter was 12 mm and the average diameter of the fifteen printed groups was 12.09 mm, which is a 0.75% variation. The relative standard deviation (RSD) for the diameter varied between 0.16% (R13) and 1.35% (R15), indicating low variability across groups. The RSD to design, which reflects the ratio of the actual to the design diameter, ranged from −0.92 (R15) to 2.08 (R13). Notably, all diameters fell within the control limits (upper control limit (UCL) = 12.39 mm, lower control limit (LCL) = 11.79 mm) as well as the variation limits recommended by FDA guidelines.

**Figure 8:**
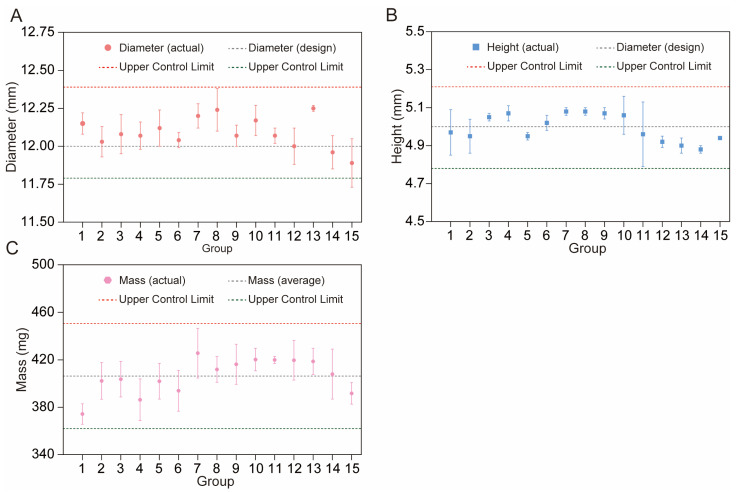
Variation in tablet diameter (**A**), height (**B**), and mass (**C**) with control limits.

The CBZ tablets exhibited an average height of 4.99 mm, with individual values ranging from 4.88 mm (R14) to 5.08 mm (R7 and R8). Height variability, reflected by RSD (0.20–3.43%, Table 1), was moderately higher than diameter variability, with the largest deviation in R11 (3.43%), likely related to printing pressure fluctuations or inconsistent material layering. The RSD to design showed balanced positive and negative deviations, indicating that while minor height variations occurred, they still fluctuated around the design height. Furthermore, all measurements remained within the control limits (UCL = 5.21 mm, LCL = 4.78 mm, Figure 8B), confirming that these fluctuations represent normal process variability rather than uncontrollable variations, and thus are acceptable for dimensional stability.

Moreover, it is noteworthy that the printing parameters did not significantly affect the macroscopic dimensions of the tablets, as evidenced by the response surface analysis of the height and dimensions (Tables S1 and S2). All complex models exhibited nonsignificant effects (*p* > 0.05), indicating that the process parameters within the tested range did not induce significant changes in the tablet size. These results matched with the consistency in geometric stability observed across tablets prepared by different parameters. However, despite the similar macroscopic dimensions, the dissolution profiles varied markedly among tablets with identical structural designs. This difference suggested that the printing parameters may adjust the microstructural properties without altering the overall dimensions, thereby influencing the drug release kinetics.

##### Mass

As shown in Table 1, the average mass of the tablets was 406.33 mg, with individual masses ranging from 374.3 mg (R1) to 425.7 mg (R7). The RSD for mass (0.71–5.17%) was the highest among the measured properties, with R14 exhibiting the largest variability (5.17%), slightly exceeding the pharmacopeial requirement of ≤5% for tablet weight variation [60,61]. This deviation was linked to R14′s parameter settings. The high printing speed (100 mm/s) likely made the nozzle’s squeezing speed and the material’s landing speed on the print platform not match. The low fan speed (20%) also made the material stay fluid longer, increasing the ability of polymer flow changes and uneven layer density. R11 showed a low 0.71% RSD, meaning high mass consistency. This may be attributed to its good parameter choices, high fan speed (100%), which quickened material solidification, low platform temperature (40 °C), which lessened excessive flow, and moderate printing speed (60 mm/s), which balanced extrusion and deposition, reducing feeding issues. Table S3 corroborated this complexity by showing that all the fitting models exhibited negative R^2^ values and *p*-values > 0.05, indicating a failure to capture the relationship between individual factors and tablet mass. Such results suggested that the mass of tablets was probably regulated by complex effects among multiple factors rather than the isolated influence of a single factor; thus, there is a need to gather and analyze a larger dataset to probe the connections between individual parameters and tablet mass.

Additionally, all mass measurements remained within the control limits (UCL = 450.67 mg, LCL = 361.99 mg, Figure 8C). Although Group R14′s variability neared the regulatory threshold, it is still within the overall process control boundaries. The low RSD of Group R11 provides evidence that the 3D printing process has the potential to produce tablets with excellent mass uniformity under ideal conditions. This consistency in mass directly correlates with stable geometric sizes, as tablet weight is inherently linked to its volume and shape. These variations represent normal process fluctuations rather than systematic errors, and such variability is considered acceptable for preliminary process characterization, as it does not compromise the validity of subsequent analyses given that core physical properties remain within controlled boundaries. Furthermore, the response surface analysis of dissolution behavior will be elaborated in Section “In Vitro Drug Release Studies of 3D-Printed Tablets”.

#### 3.4.2. In Vitro Drug Release Studies

##### Quality and Quantity of the CBZ

**Figure 9:**
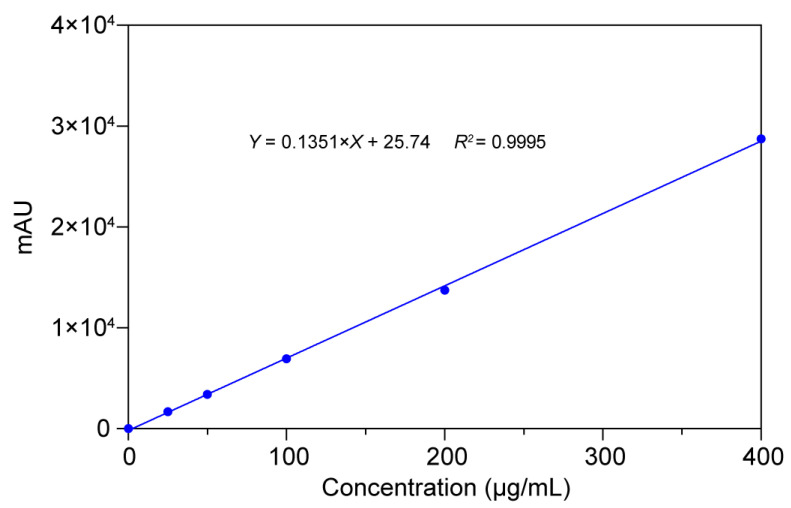
Results of calibration curves, correlation coefficients, and linear ranges of CBZ.

A CBZ calibration series (0.025–0.4 mg/mL) was established by ultrasonically dissolving 40 mg CBZ in methanol (HPLC grade), followed by sequential 1:2 volumetric dilutions. Chromatographic separation was performed using the optimized HPLC conditions detailed in Section 2.2.2. Linear regression analysis of the peak area versus analyte concentration revealed a strong correlation across the tested range (R^2^ > 0.999), as illustrated in Figure 9. This result validated the robustness of the analytical protocol for quantitative CBZ determination.

##### In Vitro Drug Release Studies of 3D-Printed Tablets

**Figure 10:**
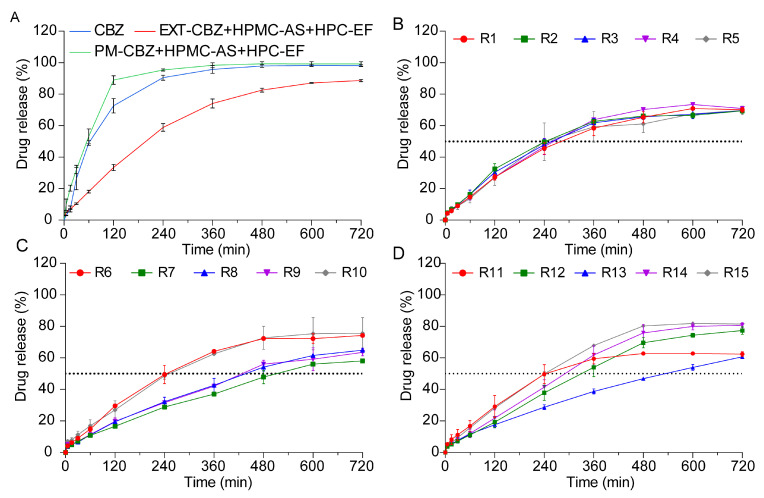
The drug release profiles of CBZ tablets prepared by different methods in pH 6.8 for 12 h. (**A**) CBZ, PM, and EXT. (**B**–**D**) R1–R15.

In vitro drug release studies demonstrated that 3D-printed tablets with identical structural designs achieved distinct release profiles due to varied printing parameters. As shown in Figure 10A, the CBZ and PM tablets rapidly disintegrate in dissolution media, leading to a faster release of CBZ compared to the printed tablets, which reached 88% and 96% CBZ released within 240 min, respectively. The EXT tablets exhibited a marked sustained-release profile of CBZ, characterized by a flat release trend over 2 h. This behavior could be attributed to ASD prepared by the HME process, which caused physical and chemical changes in the HPMC-AS, enabling the polymer to act as a diffusion barrier that slowed drug release via reduced molecular mobility and increased tortuosity [61,62].

Unlike the PM and EXT tablets, the 3D-printed tablets showed no disintegration during dissolution studies. Upon contact with the dissolution media, a steep concentration gradient formed at the interface of the 3D-printed tablets and the media. During the in vitro study, water acted as a plasticizer to reduce the system’s glass transition temperature (Tg), and the matrix transformed into a hydrogel once Tg was reached [63]. Concurrently, the HPMC-AS swelled into a hydrogel, altering the drug concentration at the media–tablet interface. Matrix imbibition of the media through the tight shell structure was the most important rate-controlling step during the dissolution study. The concentration gradients led the CBZ to diffuse from the media and tablet interface to the hydrogel, and thus into the dissolution media [47].

As shown in Figure 10B–D, despite special designs, the 3D-printed tablets did not show the same release profile of CBZ. CBZ dissolution from these tablets predominantly followed zero-order kinetics during the initial 480 min period, indicating a relatively constant release rate throughout the dissolution process. However, after 480 min, the release rate gradually declined due to drug loss within the polymer and increasing drug saturation in the dissolution medium, which collectively reduced the concentration difference driving CBZ diffusion. Although the CBZ tablets shared identical structural designs, their dissolution profiles varied significantly, a difference primarily attributed to changes in the printing parameters. Firstly, fan speed exerts a notable influence on shell compactness [64]. Lower fan speeds reduced the cooling efficiency, extending material fluidity and promoting interlayer fusion defects that increase shell porosity. Higher fan speeds would accelerate polymer solidification, yielding denser shell structures with reduced tortuosity. Platform temperature could also have an impact on shell structures [65]. High temperatures might soften the polymer, facilitate better layer adhesion but potentially cause excessive material flow that compromises shell definition. Lower temperatures may lead to incomplete layer fusion, creating micro voids in the shell that enhance drug diffusion. Specifically, the 3D-printed tablets R7, R8, R9, and 13, which exhibited lower dissolution rates, were fabricated at correspondingly slower printing speeds. This observation matched with the results in Section “Morphology Study”, where slower printing speeds resulted in less distinct shell layers, potentially altering the tablets’ structural integrity. Notably, our previous research has established that modifications to tablet shell design exert significant impacts on dissolution profiles [17]. The shell thickness significantly increases the drug dissolution rate by forming a physical diffusion barrier and reducing the effective surface area for drug release. Based on this phenomenon, the slower printing speeds observed in the R7–9 and 13 tablets likely disrupted the original shell architecture by compromising shell layer clarity, thereby reducing drug release. So, these single-factor effects highlight that the fan speed, platform temperature, and printing speed independently influence the shell porosity, layer integrity, and diffusion pathways. To fully explain how individual printing parameters modulate the shell structure, the specific effects of these parameters will be systematically elaborated in the subsequent chapters.

##### Analysis of Q_4h_ and t_50%_

Analysis of Q_4h_ and t_50%_ provided critical kinetic findings that Q_4h_ quantified the efficiency of early zero-order release, while t_50%_ reflected the transition to first-order kinetics in the 0–12 h range. As summarized in Table 2, the 3D printing parameters of the fan speed (F), platform temperature (T), and printing speed (S) could influence the CBZ release profiles. Fan speed appeared to exhibit an influence on the dissolution kinetics: lower fan speeds (20%) were associated with faster release rates (R9: Q_4h_ = 31.35%, t_50%_ = 465 min), potentially due to reduced cooling rates that enhanced the matrix porosity. Conversely, higher fan speeds (100%) tended to delay dissolution (R7: Q_4h_ = 28.72%, t_50%_ = 510 min), possibly through rapid cooling that made the polymer more compact. Platform temperature also had an effect on the release profiles: elevated temperatures (80 °C) generally accelerated dissolution (R6: t_50%_ = 245 min) by promoting polymer softening, while lower temperatures (40 °C) appeared to delay drug release (R13: t_50%_ = 530 min) [66]. Printing speed demonstrated complex effects; high speeds (100 mm/s) increased the porosity in formulations like R6 (t_50%_ = 245 min) but led to inconsistent layer fusion in R15 (t_50%_ = 240 min), likely due to combined interactions with the fan speed and temperature. Joint parameter combinations, such as R10 (20% fan speed, 80 °C, 60 mm/s), achieved optimal release kinetics (t_50%_ = 250 min), suggesting balanced thermal and mechanical processing. These trends show some directional effects. However, the exact impacts of the fan speed, platform temperature, printing speed, and their interactions need more study. Response surface methodology should be used to understand nonlinear relationships and systematically optimize process parameters.

##### Response Surface Methodology

Response surface methodology was conducted to characterize the relationships between the fan speed, platform temperature, printing speed, and t_50%_ using polynomial models with different data transformations (Table S4). The original transformation demonstrated that quadratic (R^2^ = 0.9193, *p* = 0.0027) and cubic (R^2^ = 0.9933, *p* = 0.0494) models provided superior fit compared to linear (R^2^ = 0.4614, *p* = 0.0199) and 2FI terms (R^2^ = 0.2883, *p* = 0.9529), with the cubic model capturing 99.33% of the variance despite potential overfitting. Nonlinear transformations (square-root and inverse square-root) yielded suboptimal results: square-root linear models showed negligible fit (R^2^ = −0.0063) while cubic terms (R^2^ = 0.9203) lacked significance (*p* = 0.0731); inverse square-root quadratic models (R^2^ = 0.9168, *p* = 0.0025) performed acceptably but cubic terms (R^2^ = 0.9803) were nonsignificant (*p* = 0.1388). Based on the analysis, the quadratic polynomial model under the original data transformation is recommended as the optimal fit for describing t_50%_. Specifically, this model exhibits the best balance between R^2^ = 0.9193 and statistical significance (*p* = 0.0027) among the tested models, while minimizing the overfitting risks associated with higher-order terms. Similarly, the quadratic polynomial model under the inverse square-root transformation is recommended as the optimal fit for describing Q_4h_, which exhibits the highest R^2^ (0.9019) and statistical significance (*p* = 0.0030) among all the tested models (Table S5). According to Table 3 and Table 4, both models highlighted printing speed (S) as the dominant factor (*p* < 0.005), with quadratic terms (S^2^) contributing significantly to model fit.

These results were further confirmed by the observed correlation between the printing speed and dissolution kinetics, where slower printing speeds (≤20 mm/s) were associated with reduced shell definition and correspondingly decreased drug release rates, whereas faster speeds (≥60 mm/s) accelerated dissolution. Slower printing speeds allow enough material interaction time with the layer, causing diffuse layer boundaries and compromised shell integrity that weakens the diffusion barrier function, while faster printing speeds induce higher deposition rates that lead to increased interlayer infill and more porous internal structures. These findings show that printing speed affects dissolution not just through shell clarity but also by changing the inner structure of tablets. This points to the need for optimizing both the surface and internal properties together in 3D-printed tablets, since the printing speed could influence both the shell’s definition and the pores inside. Collectively, these results emphasize the critical importance of printing speed optimization for reproducible drug release profiles, while highlighting the robustness of inverse square-root transformed models in capturing nonlinear relationships.

##### The Release Kinetics

The release kinetics of the tablets were analyzed by fitting the dissolution data to several models (zero-order, first-order, Higuchi, Korsmeyer–Peppas, and Peppas–Sahlin) across different time periods. From 0–12 h, the CBZ, PM, and groups R1–R15 showed strong first-order kinetics, which showed high correlation values (R^2^ ≥ 0.9739 in Table 5), meaning their release rates were related to the remaining drug amount, a common dissolution-driven process. While the zero-order models showed a fairly good fit for some groups, R1–R15 mainly followed first-order kinetics, where release depends on drug concentration. All formulations had good Peppas–Sahlin model fits, proving that drug diffusion and polymer erosion were key mechanisms. The model parameters k_1_ and k_2_, respectively, represent Fickian diffusion and non-Fickian release contributions. Positive k_1_ values across most groups indicated dominant diffusive transport, while the k_2_ values reflected the role of matrix erosion or swelling, even when their magnitudes varied. This contribution from k_1_ and k_2_ confirmed that the release dynamics were governed by both solute diffusion through the polymer and structural changes over time, aligning with the non-Fickian behavior observed in the Korsmeyer–Peppas analysis.

Interestingly, in the initial 0–4 h, R1–R15 showed better zero-order model fit (R^2^ ≥ 0.9847, Table 6), showing early-stage release at a nearly constant rate (zero-order), probably caused by the fast dissolving of the drug on the surface or a high early surface area compared to the volume. This contrasts with the later 0–12 h phase, where the first-order kinetics prevailed. The Peppas–Sahlin model parameters in the initial 0–4 h further clarified this behavior. Positive k_1_ values and k_2_ values across most groups suggested that while initial release appeared approximately zero-order, the kinetics still combined diffusive transport and mild matrix relaxation, collectively characterizing release mechanisms. According to the Peppas–Sahlin model, the k_1_ × t^m^ represents the Fickian diffusional contribution, whereas the k_2_ × t^2m^ represents the Case II relaxational contribution. So, R/F was applied to further understand whether diffusion or swelling controlled the drug release kinetics. The R/F equation was illustrated as follows.

$$\mathrm{Polymeric}\;\mathrm{chain}\;\mathrm{relaxation}:\;R/F=(k_{2}/k_{1})t^{m}$$

**Figure 11:**
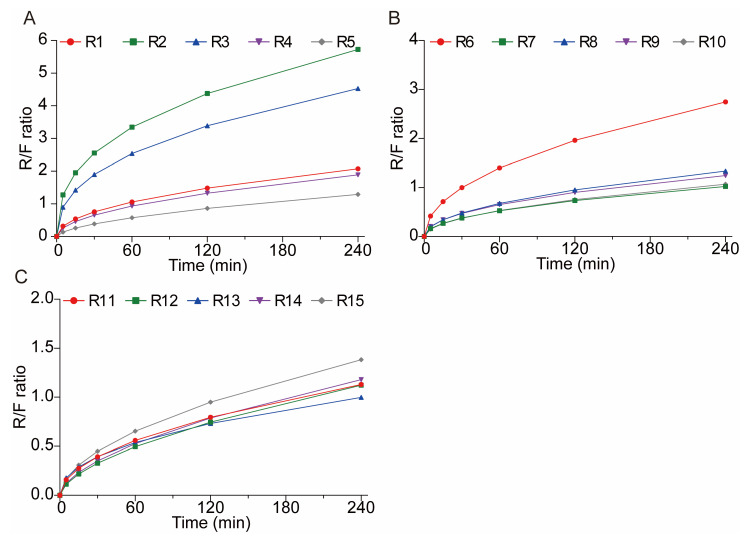
The polymeric relaxation/Fickian diffusion ratio (R/F ratio) curves of CBZ tablets. (**A**) R1–5; (**B**) R6–10; (**C**) R11–15.

As shown in Figure 11, the release mechanisms of CBZ from tablets R1–R15 exhibited stage-dependent transitions. The first 120 min of CBZ released from the 3D-printed tablets (except R1, R2, R3, and R6) were dominated by Fickian diffusion while after 120 min, the release mechanisms were dominated by the polymeric chain relaxation. The CBZ released from the remaining tablets were dominated by polymeric chain relaxation after about 60 min. The different transition times between Fickian diffusion and polymer chain relaxation are primarily driven by the printing parameters that alter the shell porosity. Porous shells allow the rapid penetration of dissolution media, limiting Fickian diffusion to approximately 60 min before hydrogel swelling becomes dominant. Meanwhile, denser shells delayed media infiltration, extending Fickian diffusion to around 120 min. Ultimately, the permeability of the tablet shell determines the balance between diffusion and polymer swelling during the drug release process.

### 3.5. Reproducibility

Groups R2, R3, and R5 were run ahead of the other groups to evaluate the reproducibility of the printing process. As demonstrated in Table 7, the variations in dimensions and weight of these three groups were relatively small, which were all within the 5% limit recommended by the FDA guidelines for manufacturing process. Additionally, the actual dimension and mass were close to the design value of the tablets, where the variation between the actual value and design value is also less than 5%. Such three batches of reproducibility study results indicated that the printing process is steady and robust, which can be used for future tablet printing. Moreover, the drug release profiles, as evidenced by the Q_4h_ and t_50%_, also exhibited low variability among these batches, further validating the reproducibility of the 3D printing process in controlling drug release behaviors. Additionally, to obtain the design space of the process parameters, the upper and lower control limit were calculated based on the data of all fifteen groups. As demonstrated in Section 3.4.1, the quality attributes of dimensions and mass were all within the calculated UCL and LCL, which indicated that the selected printing parameters were suitable for manufacturing the tablets with adequate qualities.

These results demonstrated that the 3D printing process maintains high reproducibility across critical quality attributes. Notably, the printing speed, identified as a dominant process parameter, can effectively modulate both the structural and release properties without compromising consistency. This ability to adjust parameters, along with the process’s consistent results (validated by R2, 3, and 5), shows that 3D printing can systematically optimize tablet properties. Therefore, these results establish a foundation for leveraging the printing parameters to engineer 3D-printed dosage forms with predictable and adjustable characteristics.

## 4. Conclusions

This study demonstrates the successful development of a hot-melt extrusion and FDM-3D printing approach for manufacturing CBZ-loaded sustained-release tablets, establishing a robust framework for individualized drug delivery. TGA confirmed that formulation components, including CBZ, HPMC-AS, and HPC-EF, exhibit thermal stability up to 230 °C, enabling safe processing at optimized HME and FDM-3D printing temperatures. HME turns crystalline CBZ into an amorphous form, which is proved by DSC, PLM, and PXRD. Then, DoE explored the effects of the fan speed, platform temperature, and printing speed on the tablet properties. Physical characterization revealed tight control over the dimensions and mass, with printing speed emerging as a dominant factor in dimensional accuracy: high speeds risked insufficient layer fusion, while low speeds improved uniformity. Furthermore, the release kinetics were profoundly influenced by these parameters, with the printing speed exerting the strongest effect. Response surface methodology, particularly quadratic formulations with inverse square-root transformations, accurately captured nonlinear relationships, showing that low printing speeds not only densified the polymer to delay release but also induced less distinct shell layers, whereas high speeds increased the porosity and accelerated dissolution. The platform temperature and fan speed also played roles; higher temperatures would soften the polymer to enhance release, while faster fan speeds would promote a compact structure via rapid cooling. Mechanistic analysis indicated early-stage zero-order kinetics, driven by surface drug dissolution, transitioning to first-order behavior as diffusion and matrix relaxation dominated, consistent with non-Fickian release behavior. Overall, we found that critical 3D printing parameters (especially speed) significantly affect the structure of HME-FDM 3D-printed tablets and govern their non-Fickian drug release kinetics. Through DoE, we established a parameter–structure–release relationship, providing a predictive model for optimizing 3D printing processes in pharmaceutical development. Based on this, we integrated HME with 3D printing to create a framework for personalized sustained-release drug delivery, offering a practical approach for the pharmaceutical industry to develop patient-tailored dosage forms. Current limitations include limited validation in specific polymer–drug systems. Future work will focus on the following: 1) expanding applicability to diverse materials, and 2) optimizing the printing parameters alongside structural designs to synergistically control the release profiles, advancing the clinical translation of personalized 3D-printed medications.

## Figures and Tables

**Table 1:** Box–Behnken design of 15 groups of printed tablets and the diameter, height, and mass results.

#	F	T	S	Diameter	RSD ^1^	RSD ^2^	Height	RSD ^1^	RSD ^2^	Mass	RSD ^1^
	(%)	(°C)	(mm/s)	(mm)	%		(mm)	%		(mg)	%
R1	20	40	60	12.15 ± 0.07	0.58	1.25	4.97 ± 0.12	2.41	−0.60	374.3 ± 8.6	2.30
R2	60	60	60	12.03 ± 0.10	0.83	0.25	4.95 ± 0.09	1.82	−1.00	402.3 ± 15.5	3.85
R3	60	60	60	12.08 ± 0.13	1.08	0.67	5.05 ± 0.02	0.40	1.00	403.7 ± 15.0	3.72
R4	100	80	60	12.07 ± 0.09	0.75	0.58	5.07 ± 0.04	0.79	1.40	386.3 ± 17.6	4.56
R5	60	60	60	12.12 ± 0.12	0.99	1.00	4.95 ± 0.02	0.40	−1.00	402.0 ± 15.1	3.76
R6	60	80	100	12.04 ± 0.05	0.42	0.33	5.02 ± 0.04	0.80	0.40	394.0 ± 17.3	4.39
R7	100	60	20	12.20 ± 0.08	0.66	1.67	5.08 ± 0.02	0.39	1.60	425.7 ± 21.0	4.93
R8	60	80	20	12.24 ± 0.14	1.14	2.00	5.08 ± 0.02	0.39	1.60	412.0 ± 11.0	2.67
R9	20	60	20	12.07 ± 0.07	0.58	0.58	5.07 ± 0.03	0.59	1.40	416.3 ± 17.0	4.08
R10	20	80	60	12.17 ± 0.10	0.82	1.42	5.06 ± 0.10	1.98	1.20	420.3 ± 9.5	2.26
R11	100	40	60	12.07 ± 0.05	0.41	0.58	4.96 ± 0.17	3.43	−0.80	420.0 ± 3.0	0.71
R12	60	40	100	12.00 ± 0.12	1.00	0.00	4.92 ± 0.03	0.61	−1.60	419.7 ± 16.8	4.00
R13	60	40	20	12.25 ± 0.02	0.16	2.08	4.90 ± 0.04	0.82	−2.00	418.7 ± 11.1	2.65
R14	20	60	100	11.96 ± 0.11	0.92	−0.33	4.88 ± 0.02	0.41	−2.40	408.0 ± 21.1	5.17
R15	100	60	100	11.89 ± 0.16	1.35	−0.92	4.94 ± 0.01	0.20	−1.20	391.7 ± 9.1	2.32
Average				12.09			4.99			406.33	
LCL				11.79			4.78			361.99	
UCL				12.39			5.21			450.67	

RSD ^1^: the relative variation between actual value and average value of all 15 groups. RSD ^2^: the relative variation between actual value and designed value.

**Table 2:** The results of 15 groups in Q_4h_ and t_50%_.

Group	F (%)	T (°C)	S (mm/s)	Q_4h_ (%)	t_50%_ (min)
R1	20	40	60	45.49	245
R2	60	60	60	49.63	240
R3	60	60	60	47.77	255
R4	100	80	60	46.59	260
R5	60	60	60	48.36	250
R6	60	80	100	49.17	245
R7	100	60	20	28.72	510
R8	60	80	20	32.18	430
R9	20	60	20	31.35	435
R10	20	80	60	48.36	250
R11	100	40	60	26.96	245
R12	60	40	100	37.18	335
R13	60	40	20	28.62	530
R14	20	60	100	40.86	290
R15	100	60	100	49.82	240

**Table 3:** The mathematical models of correlation between the individual factors and t_50%_.

	F	T	S	F × T	F × S	T × S	F^2^	T^2^	S^2^
*p*-value	0.8234	0.0608	0.0002	0.5857	0.1749	0.8744	1.0000	0.6001	0.0005
Significance	NS	NS	***	NS	NS	NS	NS	NS	***
Code Equation	t_50%_ = 0.21761 − 0.011099 × S + 0.00041 × S^2^								

Significance: ^NS^ *p* > 0.05, *** *p* < 0.005.

**Table 4:** The mathematical models of correlation between the individual factors and Q_4h_.

	F	T	S	F × T	F × S	T × S	F^2^	T^2^	S^2^
*p*-value	0.5282	0.0935	0.0005	0.4435	0.0911	0.3680	0.9990	0.5901	0.0005
Significance	NS	NS	***	NS	NS	NS	NS	NS	***
Code Equation	1/Sqrt(Q_4h_) = 692.34375 − 64.86486 × S + 2.29410 × S^2^								

Significance: ^NS^ *p* > 0.05, *** *p* < 0.005.

**Table 5:** The kinetic constants and correlation coefficients of different models in various groups. (Time = 0–12 h).

#	Zero-Order		First-Order		Higuchi		Korsmeyer–Peppas			Peppas–Sahlin			
	K_0_	R^2^	k	R^2^	k	R^2^	k_kp_	n	R^2^	k_1_	k_2_	m	R^2^
CBZ	0.187	0.8436	0.010	0.9982	4.470	0.9423	9.396	0.377	0.9588	3.153	−0.024	0.675	0.9911
PM	0.192	0.8036	0.014	0.9973	4.666	0.9182	14.387	0.313	0.9516	5.524	−0.073	0.595	0.9884
EXT	0.154	0.9555	0.004	0.9989	3.514	0.9896	2.303	0.570	0.9891	0.532	−0.001	0.895	0.9994
R1	0.122	0.9579	0.002	0.9938	2.797	0.9908	1.860	0.567	0.9905	0.458	−0.001	0.880	0.9995
R2	0.123	0.9341	0.002	0.9840	2.841	0.9842	2.713	0.508	0.9839	0.683	−0.002	0.829	0.9983
R3	0.122	0.9420	0.002	0.9874	2.817	0.9868	2.456	0.523	0.9863	0.616	−0.001	0.841	0.9988
R4	0.127	0.9493	0.002	0.9915	2.916	0.9850	1.933	0.568	0.9844	0.321	0.000	0.956	0.9993
R5	0.120	0.9483	0.002	0.9880	2.753	0.9869	2.082	0.546	0.9863	0.549	−0.001	0.850	0.9966
R6	0.130	0.9493	0.003	0.9928	2.984	0.9866	2.073	0.560	0.9859	0.419	−0.001	0.913	0.9990
R7	0.092	0.9889	0.001	0.9978	2.073	0.9908	0.707	0.676	0.9976	0.499	0.475	0.362	0.9977
R8	0.103	0.9874	0.002	0.9988	2.320	0.9918	0.775	0.680	0.9980	−0.699	1.100	0.319	0.9980
R9	0.102	0.9844	0002	0.9972	2.295	0.9908	0.868	0.660	0.9961	−0.406	1.062	0.318	0.9961
R10	0.132	0.9592	0.003	0.9953	3.018	0.9895	2.125	0.558	0.9896	0.515	−0.001	0.872	0.9980
R11	0.115	0.9217	0.002	0.9739	2.675	0.9787	3.073	0.477	0.9796	0.695	−0.002	0.824	0.9982
R12	0.126	0.9834	0.002	0.9966	2.811	0.9857	0.746	0.717	0.9933	−2.620	1.893	0.303	0.9938
R13	0.093	0.9909	0.001	0.9986	2.093	0.9933	0.772	0.664	0.9996	1.004	0.282	0.391	0.9997
R14	0.136	0.9757	0.003	0.9954	3.043	0.9847	0.979	0.686	0.9897	−5.017	3.366	0.270	0.9907
R15	0.143	0.9605	0.003	0.9965	3.241	0.9871	1.764	0.600	0.9875	0.296	0.000	0.980	0.9991

**Table 6:** The kinetic constants and correlation coefficients of different models in various groups. (Time = 0–4 h).

#	Zero-Order		First-Order		Higuchi		Korsmeyer–Peppas			Peppas–Sahlin			
	K_0_	R^2^	k	R^2^	k	R^2^	k_kp_	n	R^2^	k_1_	k_2_	m	R^2^
R1	0.201	0.9944	0.003	0.9982	2.552	0.9790	0.704	0.760	0.9984	1.039	0.151	0.485	0.9988
R2	0.224	0.9893	0.003	0.9970	2.856	0.9812	0.900	0.734	0.9968	0.452	0.662	0.388	0.9968
R3	0.215	0.9909	0.003	0.9976	2.744	0.9821	0.879	0.731	0.9977	0.886	0.406	0.418	0.9979
R4	0.205	0.9957	0.003	0.9982	2.589	0.9764	0.635	0.784	0.9985	0.999	0.116	0.509	0.9990
R5	0.214	0.9972	0.003	0.9949	2.674	0.9666	0.501	0.837	0.9967	0.894	0.048	0.580	0.9980
R6	0.217	0.9952	0.003	0.9978	2.734	0.9748	0.634	0.795	0.9979	0.925	0.177	0.486	0.9982
R7	0.218	0.9896	0.002	0.9924	1.653	0.9843	0.745	0.662	0.9954	1.013	0.075	0.479	0.9974
R8	0.143	0.9921	0.002	0.9957	1.831	0.9814	0.637	0.714	0.9970	0.931	0.084	0.492	0.9981
R9	0.142	0.9884	0.002	0.9926	1.831	0.9824	0.790	0.671	0.9946	1.084	0.105	0.466	0.9961
R10	0.214	0.9918	0.003	0.9923	2.735	0.9769	1.010	0.702	0.9932	1.452	0.098	0.504	0.9957
R11	0.220	0.9931	0.003	0.9963	2.810	0.9818	0.968	0.716	0.9975	1.426	0.099	0.509	0.9988
R12	0.161	0.9965	0.002	0.9944	2.018	0.9652	0.396	0.828	0.9952	0.686	0.030	0.592	0.9974
R13	0.130	0.9847	0.002	0.9899	1.697	0.9915	0.951	0.618	0.9978	1.220	0.105	0.447	0.9991
R14	0.177	0.9970	0.002	0.9953	2.222	0.9666	0.439	0.826	0.9961	0.766	0.037	0.583	0.9979
R15	0.217	0.9964	0.003	0.9970	2.739	0.9738	0.649	0.790	0.9977	1.072	0.076	0.542	0.9987

**Table 7:** Reproducibility study of 3D printing batches (R2, R3, R5) on tablet properties’ drug release profiles.

#	F	T	S	Diameter	Height	Mass	Q_4h_	t_50%_
	(%)	(°C)	(mm/s)	(mm)	(mm)	(mg)	(%)	(min)
R2	60			12.03 ± 0.10	4.95 ± 0.09	402.3 ± 15.5	49.63	240
R3		60	60	12.08 ± 0.13	5.05 ± 0.02	403.7 ± 15.0	47.77	255
R5				12.12 ± 0.12	4.95 ± 0.02	402.0 ± 15.1	48.36	250
Average				12.08	4.98	402.67	48.59	248.3
S.D.				0.05	0.06	0.91	0.95	7.6
RSD ^1^				0.37	1.16	0.23	1.96	3.06
RSD ^2^				0.38	1.15	NA	NA	NA

RSD ^1^: the relative variation between actual value and average value of all 3 groups. RSD ^2^: the relative variation between actual value and designed value.

## Data Availability

Data will be made available on request.

## Supplementary Materials

The following supporting information can be downloaded at: https://www.mdpi.com/article/10.3390/polym17152126/s1.
