# Supplementary material for: Three-Dimensional Printing of Personalized Carbamazepine Tablets Using Hydrophilic Polymers: An Investigation of Correlation Between Dissolution Kinetics and Printing Parameters
Source: Polymers (Basel). 2025 Aug 1;17(15):2126. doi: 10.3390/polym17152126 (PMC12349063; doi:10.3390/polym17152126)
Supplement: Supplementary file 1 [file polymers-17-02126-s001.zip › polymers-3748221-supplementary.pdf]

Table S1. The fitting models of the correlation between individual factors and Diameter.

Model	Source	R^2	*p-value*
Original	Linear	-0.0069	0.4424
	2FI	-0.2675	0.8618
	Quadratic	-0.1246	0.3609
	Cubic	0.0523	0.4603
Square Root	Linear	-0.0063	0.4412
	2FI	-0.2673	0.8627
	Quadratic	-0.1225	0.3596
	Cubic	0.0568	0.4590
Inverse Sqrt	Linear	-0.0050	0.4387
	2FI	-0.2668	0.8644
	Quadratic	-0.1183	0.3570
	Cubic	0.0659	0.4566

Table S2. The fitting models of the correlation between individual factors and Height.

Model	Source	R^2	*p-value*
Original	Linear	0.2265	0.1268
	2FI	0.1098	0.6805
	Quadratic	0.5645	0.0931
	Cubic	0.4063	0.6933
Square Root	Linear	0.2267	0.1266

	2FI	0.1108	0.6789
	Quadratic	0.5653	0.0929
	Cubic	0.4104	0.6906
Inverse Sqrt	Linear	0.2271	0.1263
	2FI	0.1127	0.6757
	Quadratic	0.5671	0.0925
	Cubic	0.4186	0.6851

Table S3. The fitting models of the correlation between individual factors and Mass.

Model	Source	R^2	*p-value*
Original	Linear	-0.0379	0.5048
	2FI	-0.3552	0.9323
	Quadratic	-0.2545	0.3952
	Cubic	-0.2864	0.5470
Square Root	Linear	-0.0411	0.5116
	2FI	-0.3593	0.9321
	Quadratic	-0.2603	0.3966
	Cubic	-0.3314	0.5612
Inverse Sqrt	Linear	-0.0477	0.5256
	2FI	-0.3675	0.9316
	Quadratic	-0.2724	0.3996
	Cubic	-0.4234	0.5893

Table S4. The fitting models of the correlation between individual factors and $t_{50\%}$.

Model	Source	R^2	*p-value*
Original	Linear	0.4614	0.0199
	2FI	0.2883	0.9529
	Quadratic	0.9193	0.0027
	Cubic	0.9933	0.0494
Square Root	Linear	-0.0063	0.0225
	2FI	0.4486	0.9477
	Quadratic	0.2736	0.0024
	Cubic	0.9203	0.0731
Inverse Sqrt	Linear	0.4179	0.0299
	2FI	0.2494	0.9090
	Quadratic	0.9168	0.0025
	Cubic	0.9803	0.1388

Table S5. The fitting models of the correlation between individual factors and Q_{4h}.

Model	Source	R^2	*p-value*
Original	Linear	0.2980	0.0779
	2FI	0.1439	0.7974
	Quadratic	0.8898	0.0036
	Cubic	0.9870	0.0702
Square Root	Linear	0.3133	0.0697
	2FI	0.1516	0.8239
	Quadratic	0.8946	0.0033

	Cubic	0.9896	0.0588
Inverse Sqrt	Linear	0.3441	0.0552
	2FI	0.1722	0.8673
	Quadratic	0.9019	0.0030
	Cubic	0.9936	0.0390
